# Effects of Zinc Compounds on the Enzymatic Activities of Lysozyme and Peroxidase and Their Antifungal Activities

**DOI:** 10.1007/s12011-024-04110-x

**Published:** 2024-02-20

**Authors:** Yongdae Kim, Ji-Youn Chang, Yoon-Young Kim, Jae Wook Lee, Hong-Seop Kho

**Affiliations:** 1 Department of Oral Medicine and Oral Diagnosis, School of Dentistry and Dental Research Institute, Seoul National University, 101 Daehak-Ro, Jongno-Gu, Seoul, 03080 South Korea; 2 Natural Product Research Center, Korea Institute of Science and Technology (KIST), Gangneung Institute, 679 Saimdang-Ro, Gangneung, 25451 South Korea; 3 National University of Science and Technology, Daejeon, South Korea; 4 Institute of Aging, Seoul National University, Seoul, South Korea

**Keywords:** Zinc, Saliva, Lysozyme, Peroxidase, Antifungal activity

## Abstract

This study aimed to investigate the effects of zinc compounds on the enzymatic activities of lysozyme, peroxidase, and the glucose oxidase–mediated peroxidase (GO-PO) system and their antifungal activities. Four different zinc compounds (zinc chloride, gluconate, lactate, and sulfate) were incubated with hen egg-white lysozyme (HEWL), bovine lactoperoxidase (bLPO), the GO-PO system, and human unstimulated whole saliva in solution and on a hydroxyapatite surface. Enzymatic activities of lysozyme, peroxidase, and the GO-PO system were measured through the hydrolysis of *Micrococcus lysodeikticus*, oxidation of fluorogenic 2′,7′-dichlorofluorescin, and glucose assay, respectively. Interactions between zinc and enzymes were analyzed by surface plasmon resonance (SPR). The minimum inhibitory concentration (MIC) and candidacidal activities of zinc compounds were examined against three *Candida albicans* strains. Zinc gluconate and sulfate significantly increased the enzymatic activities of salivary lysozyme in the solution assay and of HEWL and salivary lysozyme on the hydroxyapatite surface. However, all examined zinc compounds significantly decreased the enzymatic activities of bLPO and salivary peroxidase in solution and on the surface. SPR analyses revealed binding of zinc to lysozyme and peroxidase, with affinity differing according to the zinc compounds. The MIC of zinc compounds against *C. albicans* was 1.0–2.4 mM. Candidacidal activities were 17.7–38.8% and 23.7–47.0% at 1.0 and 10 mM concentrations, respectively. In conclusion, zinc compounds enhanced lysozyme activity but inhibited peroxidase activity. Zinc compounds exhibited concentration-dependent candidacidal activity against *C. albicans*. Zinc compounds are potential therapeutic agents for oral health, especially for geriatric patients.

**Supplementary Information:**

The online version contains supplementary material available at 10.1007/s12011-024-04110-x.

## Introduction

Zinc is an essential trace element and an important micronutrient for the human body. It is involved in innate and adaptive immune function, regulation of inflammation and oxidative activity, and wound healing and tissue regeneration [1–3]. It contributes to the activation of various enzymes and proteins required for the regulation of protein, lipid, nucleic acid metabolism, and gene transcription [1, 4]. Zinc has diverse antimicrobial activities [5–9]. Because of zinc’s biological activities, its compounds have served as therapeutic agents for skin diseases and as adjuvant agents for neurological diseases and/or cancers [2, 4, 10]. Furthermore, zinc compounds are included in skin care products and dietary supplements as they are safe to use [4, 11].

Zinc is present in saliva, teeth, and dental plaque in the oral cavity and may play certain roles in the de- and re-mineralization of dental hard tissue [12]. Zinc modulates gingival/periodontal inflammation by regulating plaque formation [13, 14] and antibacterial activity [5–7]. Zinc also modulates innate immune cell function, immune function of host cells [1–3], and activity and expression of matrix metalloproteinases [3]. In addition, zinc is an effective oral malodor reducing agent. Therefore, zinc has been included as a component in toothpastes and mouth rinses to augment anti-caries and anti-gingivitis activities and to reduce oral malodor [7, 15, 16]. However, limited information is present on its antifungal activity. Zinc compounds reportedly exhibit antifungal properties against food-related [9] and plant-related fungi [17], but information on their antifungal effects against oral *Candida*, which is important following the increase in the aging population with a high prevalence of oral candidiasis, remains limited [7, 8].

When developing oral health care products using zinc compounds, the zinc compounds may interact with the antimicrobial enzymes present in the products. In addition, oral health care products including zinc compounds may interact with antimicrobial enzymes present in saliva [18]. Lysozyme and peroxidase are representative molecules among these antimicrobial enzymes, and compounds of animal origin have usually been utilized in oral health care products [19]. For products utilizing peroxidase, the glucose oxidase–mediated peroxidase (GO-PO) system is used to mimic the natural peroxidase system [20]. The molecular interactions in the oral cavity occur in the solution phase, such as saliva, and on the surface phase, such as teeth. When surfaces, such as the hydroxyapatite of tooth enamel, adsorb a molecule, its molecular structure changes conformationally [21, 22]. Therefore, the result of the molecular interactions on the tooth surface may differ from those in the solution, such as saliva or rinsing solution [23–25]. Therefore, understanding the effect of zinc compounds on the enzymatic activities of salivary antimicrobials in both the solution and surface phases is essential for the development of oral health therapeutic agents containing zinc compounds [18].

The suffering from oral side effects due to multimorbidity and polypharmacy in aged and super-aged society has become a big social and individual burden [26, 27]. The sufferings include dry mouth, root caries, periodontal inflammation, oral mucosal ulcers, oral pain, increased and recurrent fungal infections, and oral malodor that could isolate elderly from society [26–28]. Therefore, the need for topical agents that can be easily, repeatedly, and safely applied by these populations to prevent or treat oral side effects with minimized systemic physical burden is increasing. Considering zinc’s biological properties, it is supposedly worth developing as an effective topical agent. Therefore, it is essential to explore the interaction between zinc and salivary antimicrobial molecules, and its antifungal activity. Further information on the differences between several zinc compounds and appropriate concentrations is warranted for the development of effective topical therapeutic agents for oral diseases and conditions.

This study aimed to investigate the effects of zinc compounds on the enzyme activities of lysozyme, peroxidase, and GO-PO systems along with their antifungal activities against *Candida albicans*.

## Materials and Methods

### Zinc Compounds, Lysozyme, Peroxidase, and the GO-PO System

Four different zinc compounds were used in this study: zinc chloride (ZnCl_2_, M.W. 136.3), zinc gluconate (ZnC_12_H_22_O_14_, M.W. 455.7), zinc lactate (ZnC_6_H_10_O_6_·2H_2_O, M.W. 279.6), and zinc sulfate (ZnSO_4_·7H_2_O, M.W. 287.6). Zinc chloride, gluconate, and sulfate were obtained from Sigma-Aldrich Chemical Co. (St. Louis, MO, USA). Zinc lactate was obtained from Jindan Lactic Acid Technology Co. (Henan, China). Concentrations of 3.6 and 7.2 mM were prepared for each zinc compound using simulated salivary buffer (1.95 mM potassium phosphate dibasic, 16 mM potassium chloride, 14.5 mM sodium chloride, 0.96 mM calcium chloride dihydrate, pH 5.5). The concentrations of zinc compounds used in the experiments were determined based on previous studies on oral health therapeutic agents [6, 7, 16, 29]. The pH was set at 5.5 for the solubility of the zinc compounds.

Hen egg-white lysozyme (HEWL) at final concentration of 30 μg/mL and bovine lactoperoxidase (bLPO) at final concentration of 25 μg/mL (Sigma-Aldrich Chemical Co.) were used as lysozyme and peroxidase sources, respectively. The reagents in the glucose assay kit (Sigma-Aldrich Chemical Co.) were used as a source for the GO-PO system. All reagents were dissolved in the same simulated salivary buffer.

### Collection of Human Saliva

Four healthy adults participated in this study (two men and women; age, 31.0 ± 5.2 years). The unstimulated whole saliva (UWS) samples of the participants were collected between 9 a.m. and 11 a.m. to minimize circadian variations. None of the participants had medical history of severe illnesses or medications affecting salivary secretion during the previous 3 months. The participants were required to refrain from eating, drinking, and brushing for at least 1 h before saliva collection. UWS was collected using the spitting method and placed in a chilled tube. The saliva sample was centrifuged at 3500 × *g* for 20 min at 4 °C. The same amounts of clarified supernatants from the participants were pooled and used immediately for analysis as a source of lysozyme and peroxidase. The Institutional Review Board of Seoul National University Dental Hospital approved the research protocols (20 Jul. 2022 / No. CRI22013), and informed consent was obtained from all participants.

### Enzymatic Activities of Lysozyme and Peroxidase

Lysozyme activity was determined through the hydrolysis of fluorescein-labeled *Micrococcus lysodeikticus* using the EnzCheck Lysozyme assay kit (Molecular Probes, Eugene, OR, USA). Peroxidase activity was determined through the oxidation of fluorogenic 2′,7′-dichlorofluorescein to fluorescent dichlorofluorescein in the presence of peroxidase and hydrogen peroxidase. Enzymatic activity was measured using a fluorescence microplate reader (BioTek instruments Inc., Winooski, VT, USA) and detailed methods have been previously described [30]. The enzymatic activities of lysozyme and peroxidase were measured eight times in duplicate.

#### *Influence of Zinc Compounds on the Enzymatic Activities in Solution*

The effects of zinc compounds on the enzymatic activity of HEWL and salivary lysozyme (bLPO and salivary peroxidase) in solution were examined by incubating 500 μL of two different zinc compound concentrations with 500 μL of HEWL (bLPO) or clarified UWS for 30 min at room temperature (RT). Detailed methods have been previously described [30]. The incubated buffer with HEWL (bLPO) or clarified UWS served as a control. An incubated mixture of each zinc compound with the buffer or an incubated buffer alone served as the blank.

#### Influence of Zinc Compounds on the Enzymatic Activities of the Hydroxyapatite Surface

Ceramic hydroxyapatite beads (Macro-prep HA type I, Bio-Rad, Hercules, CA, USA) were used for surface analysis. Hydroxyapatite beads (10 mg) were used in each assay. For the surface assay of lysozyme (peroxidase), 300 μL of two different zinc compound concentrations was preincubated with 300 μL of HEWL (bLPO) or clarified UWS for 30 min at RT. Subsequently, hydroxyapatite beads were incubated with 600 μL of each zinc compound-lysozyme (-peroxidase) mixture for 30 min at RT and then washed five times with buffer to remove unbound molecules. These beads were used for measuring enzymatic activity. Hydroxyapatite beads incubated with HEWL (bLPO) or clarified UWS served as a control. Equal amounts of hydroxyapatite beads incubated with zinc compounds or an incubated buffer alone served as blanks. Detailed methods have been previously described [30].

### Enzymatic Activity of the GO-PO System

The glucose oxidase and peroxidase reagents in the glucose assay kit were divided into two parts, one dissolved in the simulated salivary buffer and the other dissolved in the buffer containing each zinc compound of two concentrations, and preincubated for 30 min at RT. The enzymatic activity of the GO-PO system was determined by oxidized o-dianisidine production using samples with known glucose concentrations (0.04 mg/mL). Detailed methods have been previously described [24].

### Interaction Analyses Between Zinc Compounds and Lysozyme or Peroxidase Using Surface Plasmon Resonance

For binding analyses, a surface plasmon resonance (SPR) apparatus (iMSPR-ProX, Icluebio, Sungnam, South Korea) was used. N-Hydroxysuccinimide (NHS), 1-ethyl-3-(3-dimethylaminopropyl) carbodiimide hydrochloride (EDC), ethanolamine, sodium acetate buffer, and 10 mM HBST buffer (10 mM HEPES, 150 mM NaCl with 0.005% tween 20, pH 7.4) were used as received (Icluebio).

At the time of analyses, bLPO importation into South Korea was prohibited and bLPO was unavailable; therefore, horseradish peroxidase (HRP) was used instead of bLPO.

#### Immobilization of Protein

The HC1000M sensor chips (XanTec bioanalytics GmbH, Dusseldorf, Germany) were activated for 7 min with 240 μL of a mixture of 0.26 M EDC and 0.10 M NHS. Then 298 μL of HEWL (50 μg/mL dissolved in 5 mM sodium acetate buffer, pH 5.0) or 198 μL of HRP (200 μg/mL dissolved in 5 mM sodium acetate buffer, pH 4.0) was covalently coupled to the NHS-activated sensor chip matrix for more than 20 min (10 min in HRP) with a flow rate of 10 μL/min. Residual reactive groups were blocked by adding 160 μL of 1.0 M ethanolamine. Finally, the sensor surface was washed twice with 130 μL of 10 mM glycine–HCl buffer (pH 2.5). Under these conditions, HEWL and HRP immobilization onto the HC1000M sensor chips yielded approximately 8000 and 2125 resonance units, respectively.

#### Adsorption Experiment

Zinc compounds were solubilized in 10 mM HBST buffer to prepare solutions in five concentrations for HEWL (0.5, 2.0, 8.0, 35, and 70 μM) and six concentrations for HRP (25, 50, 100, 200, 400, and 800 μM) experiments. The SPR signals resulting from the adsorption of zinc compounds on immobilized HEWL or HRP surfaces were measured at different zinc compound concentrations. The adsorption amounts of the zinc compounds on the sensor surfaces bearing HEWL or HRP were determined by injecting the zinc compound solutions into the flow cell. The HBST buffer (pH 7.4) was used as the running buffer at a flow rate of 10 μL/min. The sensor chip was regenerated using 130 μL injections of 10 mM glycine–HCl buffer (pH 2.5) at a flow rate of 30 μL/min for 1 min for HEWL and 3 min for HRP. The complete experiment was repeated twice.

Association (*K*_A_) and dissociation equilibrium constants (*K*_D_) for the adsorption of different zinc compounds to immobilized HEWL or HRP proteins were obtained as follows: the adsorption (*k*_a_) and desorption (*k*_d_) rate constants were determined using the software provided by the manufacturer (Tracedrawer, Ridgeview Instruments, Uppsala, Sweden). Then, *K*_D_ was calculated as the ratio of *k*_d_ to *k*_a_.

### Antifungal Activity of Zinc Compounds

Minimum inhibitory concentration (MIC) and candidacidal assays were performed to examine the antifungal activity of zinc compounds. *Candida albicans* ATCC strains 10,231, 11,006, and 18804 were used in the experiments.

#### Minimum Inhibitory Concentration Assay of Zinc Compounds

The MIC assay against *C. albicans* strains was performed using the standard broth microdilution method in YM medium according to the reference method [31]. Equal volumes of candidal cell inoculum (1 × 10^5^ cells/mL) were added into each well of microtiter plates containing different zinc compound concentrations, with final concentrations of 10–0.1 mM. The plate was incubated overnight at 37℃, and measured at 550 nm. The MIC endpoint was determined as the lowest concentration that significantly decreased growth below the control levels. The experiment was performed five times in duplicates for each zinc compound and *Candida* strain.

#### Candidacidal Activity of Zinc Compounds

Candidacidal activities of zinc compounds were investigated at final concentrations of 0.01 μM, 0.1 μM, 1 μM, 10 μM, 100 μM, 1 mM, and 10 mM in preliminary experiments. Because the candidacidal activities at 0.01 μM and 0.1 μM were negligible, concentrations from 1 μM to 10 mM were used for the study.

One *C. albicans* colony was inoculated into 10 mL Sabouraud dextrose broth and incubated with shaking at 37℃ for 18 h. Cells were harvested, washed, and resuspended to 1 × 10^5^ cells/mL in the buffer. Cell suspension (20 μL) was added to 40 μL of zinc compounds. The mixture samples were incubated with shaking at 37℃ for 1 h. After incubation, the mixtures were diluted tenfold, and 50 μL of the diluted cells was plated onto Sabouraud dextrose agar plates in triplicate and grown overnight at 37℃. The percentage loss of cell viability (candidacidal activity) was determined by comparing the number of colonies on the experimental (with zinc compounds) and control (without zinc compounds) plates. The experiments were performed six times.

### Statistics

The Wilcoxon signed rank test was used to analyze the statistical differences between the experimental (with zinc compounds) and the control groups (without zinc compounds). The Kruskal–Wallis test was used to analyze statistical differences among zinc compounds and the Mann–Whitney *U* test was used for post hoc analysis. *P*-value less than 0.05 was considered statistically significant.

## Results

### Effects of Zinc Compounds on the Enzymatic Activities of Lysozyme

The effects of zinc compounds on the enzymatic activities of HEWL and salivary lysozyme are shown in Table 1 and 2, and Supplementary Table 1.1 and 1.2.

**Table 1:** Effect of zinc compounds on the enzymatic activities of hen egg-white lysozyme and whole salivary lysozyme in solution

Source of lysozyme	Ratio of lysozyme activity (%, median [interquartile range] (mean), *n* = 8)								
	Zinc (mM)	Zinc chloride		Zinc gluconate		Zinc lactate		Zinc sulfate	
		Ratio	*P* value	Ratio	*P* value	Ratio	*P* value	Ratio	*P* value
HEWL	3.6	102.1 [99.3–104.8] (102.3)	.093	100.7 [98.6–104.6] (101.2)	.401	101.4 [99.7–103.8] (101.4)	.208	96.2 [94.6–101.2] (97.9)	.263
	7.2	104.2 [97.8–105.7] (102.3)	.093	98.7 [95.9–100.0] (98.5)	.123	100.8 [99.7–104.6] (101.6)	.263	98.9 [96.9–101.7] (99.1)	.327
Human saliva	3.6^§^	101.3 [99.4–105.5] (101.8)^a,b^	.263	111.3 [108.8–114.2] (111.5)^a,c,d^	.012*	101.4 [98.7–104.6] (101.6)^c,e^	.208	130.6 [124.1–146.9] (133.9)^b,d,e^	.012*
	7.2^§^	101.6 [100.6–102.6] (100.6)^a,b^	.161	118.3 [106.5–122.6] (115.3)^a,c,d^	.012*	102.9 [100.1–104.5] (102.5)^c,e^	.050	154.0 [138.8–167.3] (153.8)^b,d,e^	.012*

*HEWL* hen egg-white lysozyme

Ratio, percent changes in enzymatic activities in samples with zinc compounds compared with those without zinc compounds

The Wilcoxon signed rank test was used to analyze differences between the enzymatic activities of samples with and without zinc compounds. *P < 0.05

The Kruskal–Wallis test was used to analyze differences in the ratio among zinc compounds at the same concentration. ^§^*P* < 0.05

The Mann–Whitney *U* test was used for post hoc analysis. Pairs of the same letter denote a significant difference at the same concentration. *P* < 0.05

**Table 2:** Effect of zinc compounds on enzymatic activities of hen egg-white lysozyme and whole salivary lysozyme on the hydroxyapatite surface

Source of lysozyme	Ratio of lysozyme activity (%, median [interquartile range] (mean), *n* = 8)								
	Zinc (mM)	Zinc chloride		Zinc gluconate		Zinc lactate		Zinc sulfate	
		Ratio	*P* value	Ratio	*P* value	Ratio	*P* value	Ratio	*P* value
HEWL	3.6^§^	98.8 [98.3–101.4] (99.0)^a,b^	.484	105.7 [103.8–107.7] (105.1)^a,c,d^	.017*	99.7 [98.1–102.0] (99.6)^c,e^	.779	113.9 [110.1–115.5] (113.4)^b,d,e^	.012*
	7.2^§^	108.3 [103.0–111.4] (107.5)^a^	.017*	104.2 [101.8–109.8] (105.6)^b^	.012*	103.8 [100.6–110.7] (105.2)^c^	.012*	119.2 [116.4–121.2] (119.0)^a,b,c^	.012*
Human saliva	3.6^§^	96.0 [93.3–101.9] (97.6)^a,b^	.263	108.7 [104.1–120.1] (111.0)^a^	.012*	102.0 [91.1–110.3] (101.1)^c^	.889	125.0 [109.5–134.9] (122.5)^b,c^	.017*
	7.2^§^	97.0 [95.9–99.8] (100.1)^a,b^	.208	117.8 [107.2–133.2] (119.6)^a,c,d^	.012*	106.0 [102.0–109.2] (106.1)^c^	.046*	125.3 [123.0–130.7] (125.7)^b,d^	.012*

*HEWL* hen egg-white lysozyme

Ratio, percent changes in enzymatic activities in samples with zinc compounds compared with those without zinc compounds

The Wilcoxon signed rank test was used to analyze differences between the enzymatic activities of samples with and without zinc compounds. *P < 0.05

The Kruskal–Wallis test was used to analyze differences in the ratio among zinc compounds at the same concentration. ^§^*P* < 0.05

The Mann–Whitney *U* test was used for post hoc analysis. Pairs of the same letter denote a significant difference at the same concentration. *P* < 0.05

In the solution assay, the zinc compounds used did not significantly affect the enzymatic activity of HEWL. However, they exhibited a different pattern in the salivary lysozyme. Zinc gluconate and sulfate significantly increased the enzymatic activity and effect of salivary lysozyme was concentration-dependent. The increase rate of zinc sulfate was greater than that of zinc gluconate. Zinc sulfate at 7.2 mM showed an increase of more than 50% (Table 1 and Supplementary Table 1.1).

On the surface assay, the enzymatic activities increased significantly in both HEWL and salivary lysozyme. For HEWL, zinc compounds at a concentration of 7.2 mM significantly increased the enzymatic activities in all four types. Zinc gluconate and sulfate significantly increased enzymatic activities at both concentrations in HEWL and salivary lysozyme, and the increase rates were significantly higher in zinc sulfate than in zinc gluconate (Table 2 and Supplementary Table 1.2).

### Effects of Zinc Compounds on the Enzymatic Activities of Peroxidase

The effects of zinc compounds on the enzymatic activity of peroxidase differed from those of lysozyme. Zinc compounds decreased the enzymatic activities of peroxidase (Table 3 and 4, and Supplementary Table 2.1 and 2.2).

**Table 3:** Effect of zinc compounds on the enzymatic activities of bovine lactoperoxidase and whole salivary peroxidase in solution

Source of peroxidase	Ratio of peroxidase activity (%, median [interquartile range] (mean), *n* = 8)								
	Zinc (mM)	Zinc chloride		Zinc gluconate		Zinc lactate		Zinc sulfate	
		Ratio	*P* value	Ratio	*P* value	Ratio	*P* value	Ratio	*P* value
bLPO	3.6	93.1 [89.7–97.6] (93.9)^a^	.021*	89.6 [85.7–98.5] (91.6)^b^	.036*	95.1 [90.9–95.7] (93.9)	.050	102.2 [94.3–103.0] (99.5)^a,b^	.674
	7.2	93.7 [73.8–98.2] (88.7)	.012*	87.1 [74.8–96.2] (86.4)	.025*	86.3 [78.7–90.3] (85.4)	.012*	93.3 [92.2–97.1] (94.4)	.012*
Human saliva	3.6^§^	84.4 [83.7–85.9] (84.6)^a^	.012*	93.1 [89.1–94.8] (92.5)^a,b,c^	.012*	85.7 [84.9–87.4] (86.0)^b^	.012*	86.6 [82.4–89.0] (85.9)^c^	.012*
	7.2^§^	76.2 [75.0–80.4] (76.9)^a,b^	.012*	89.0 [86.5–90.6] (88.5)^a,c,d^	.012*	82.1 [79.7–83.4] (81.5)^b,c,e^	.012*	75.4 [74.3–81.4] (77.2)^d,e^	.012*

*bLPO* bovine lactoperoxidase

Ratio, percent changes in enzymatic activities in samples with zinc compounds compared with those without zinc compounds

The Wilcoxon signed rank test was used to analyze differences between the enzymatic activities of samples with and without zinc compounds. *P < 0.05

The Kruskal–Wallis test was used to analyze differences in the ratio among zinc compounds at the same concentration. ^§^*P* < 0.05

The Mann–Whitney *U* test was used for post hoc analysis. Pairs of the same letter denote a significant difference at the same concentration. *P* < 0.05

**Table 4:** Effect of zinc compounds on the enzymatic activities of bovine lactoperoxidase and whole salivary peroxidase on the hydroxyapatite surface

Source of peroxidase	Ratio of peroxidase activity (%, median [interquartile range] (mean), n = 8)								
	Zinc (mM)	Zinc chloride		Zinc gluconate		Zinc lactate		Zinc sulfate	
		Ratio	*P* value	Ratio	*P* value	Ratio	*P* value	Ratio	*P* value
bLPO	3.6^§^	95.0 [94.9–96.6] (95.4)^a,b^	.012*	101.8 [96.2–105.3] (101.3)^a^	.327	96.5 [96.0–97.6] (96.6)^c^	.011*	102.5 [97.4–104.9] (101.5)^b,c^	.484
	7.2^§^	88.1 [86.0–91.7] (88.7)^a,b^	.012*	97.3 [94.7–99.3] (96.7)^a,c,d^	.017*	93.8 [93.2–96.7] (94.7)^b,c^	.012*	93.5 [88.8–97.6] (93.4)^d^	.025*
Human saliva	3.6	54.1 [47.4–61.7] (54.8)	.012*	60.1 [56.7–61.1] (59.1)	.012*	56.1 [53.4–62.6] (57.8)	.012*	55.1 [53.7–57.2] (55.4)	.012*
	7.2^§^	44.7 [40.3–49.1] (45.0)^a,b,c^	.012*	45.8 [43.2–48.3] (46.0)^a^	.012*	44.0 [39.1–52.9] (45.7)^b^	.012*	42.3 [39.5–48.4] (43.5)^c^	.012*

*bLPO* bovine lactoperoxidase

Ratio, percent changes in enzymatic activities in samples with zinc compounds compared with those without zinc compounds

The Wilcoxon signed rank test was used to analyze differences between the enzymatic activities of samples with and without zinc compounds. *P < 0.05

The Kruskal–Wallis test was used to analyze differences in the ratio among zinc compounds at the same concentration. ^§^*P* < 0.05

The Mann–Whitney *U* test was used for post hoc analysis. Pairs of the same letter denote a significant difference at the same concentration. *P* < 0.05

In the solution assay, except zinc lactate (*P* = 0.050) and sulfate at 3.6 mM concentration, all zinc compounds decreased the enzymatic activity of bLPO. All zinc compounds also decreased the enzymatic activities of salivary peroxidase at 3.6 and 7.2 mM concentrations (Table 3 and Supplementary Table 2.1).

On the surface assay, the enzymatic activities of bLPO and salivary peroxidase were decreased in the presence of zinc compounds under most experimental conditions. In particular, zinc compounds at a concentration of 7.2 mM decreased more than 50% of the enzymatic activity of salivary peroxidase (Table 4 and Supplementary Table 2.2).

### Effects of Zinc Compounds on the GO-PO System

Table 5 and Supplementary Table 3 show the effects of zinc compounds on the GO-PO system. Only zinc lactate at a concentration of 7.2 mM significantly reduced the enzymatic activity of the GO-PO system (*P* < 0.043). The other zinc compounds showed no significant differences.

**Table 5:** Influence of zinc compounds on the enzymatic activities of glucose oxidase-mediated peroxidase

Zinc compounds	Ratio of glucose oxidase-mediated peroxidase activity (%, median [interquartile range] (mean), *n* = 5)			
	With zinc compound			
	3.6 mM^§^	*P* value	7.2 mM^§^	*P* value
Zinc chloride	98.3 [97.2–100.4] (98.7)^a^	.225	100.3 [100.0–100.6] (100.3)^a^	.144
Zinc gluconate	101.4 [99.7–101.8] (100.9)	.225	101.3 [99.9–101.7] (100.9)^b^	.138
Zinc lactate	99.6 [98.3–99.9] (99.2)	.068	99.2 [98.0–99.7] (98.9)^a,b,c^	.043*
Zinc sulfate	100.9 [99.9–101.1] (100.6)^a^	.138	101.1 [100.2–102.0] (101.1)^c^	.078

The Wilcoxon signed rank test was used to analyze differences between the enzymatic activities of samples with and without zinc compounds. *P < 0.05

The Kruskal–Wallis test was used to analyze differences in the ratio among zinc compounds at the same concentration. ^§^*P* < 0.05

The Mann–Whitney *U* test was used for post hoc analysis. Pairs of the same letter denote a significant difference at the same concentration. *P* < 0.05

### SPR Analyses

**Fig. 1:**
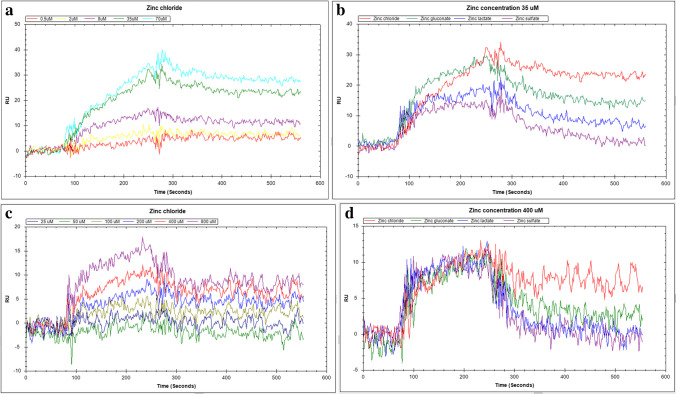
Interaction analyses between zinc compounds and hen egg-white lysozyme or horseradish peroxidase using surface plasmon resonance. **a** Interactions between zinc chloride at different concentrations and hen egg-white lysozyme, **b** interactions between different zinc compounds at 35 μM and hen egg-white lysozyme, **c** interactions between zinc chloride at different concentrations and horseradish peroxidase, and **d** interactions between different zinc compounds at 400 μM and horseradish peroxidase. RU, resonance units

Figure 1 a and 1 c show the increased interactions between zinc chloride and HEWL or HRP with increasing zinc chloride concentrations. The same phenomena were observed for all other zinc compounds used (data not shown). The rate and equilibrium constants as a measure of HEWL or HRP affinity for each zinc compound were analyzed (Table 6). The values of *k*_a_ were zinc sulfate > lactate > chloride > gluconate and those of *k*_d_ were zinc sulfate > lactate > gluconate > chloride. The values of *K*_D_ for zinc chloride were the lowest in both HEWL and HRP, indicating the highest affinity. Figure 1 b and d show similar results; differences in the interactions according to zinc compounds at concentrations of 35 μM for HEWL and 400 μM for HRP.

**Table 6:** Interaction analyses between zinc compounds and lysozyme or peroxidase using surface plasmon resonance

	*k*_a_ (M^−1^ s^−1^)	*k*_d_ (s^−1^)	*K*_D_ (M)
Lysozyme (hen egg-white lysozyme)			
Zinc chloride	3.38 × 10^2^	6.14 × 10^−4^	1.83 × 10^−6^
Zinc gluconate	3.22 × 10^2^	22.00 × 10^−4^	6.84 × 10^−6^
Zinc lactate	7.98 × 10^2^	55.00 × 10^−4^	7.19 × 10^−6^
Zinc sulfate	8.31 × 10^2^	61.45 × 10^−4^	8.45 × 10^−6^
Peroxidase (horseradish peroxidase)			
Zinc chloride	3.59 × 10^1^	2.06 × 10^−3^	5.74 × 10^−5^
Zinc gluconate	3.13 × 10^1^	3.95 × 10^−3^	1.26 × 10^−4^
Zinc lactate	4.45 × 10^1^	7.22 × 10^−3^	1.62 × 10^−4^
Zinc sulfate	9.08 × 10^1^	2.77 × 10^−2^	3.05 × 10^−4^

*k*_*a*_, association rate constant; *k*_*d*_, dissociation rate constant; *K*_*D*_, equilibrium dissociation constant

### Antifungal Activities of Zinc Compounds

#### MIC Assay

Table 7 shows the MICs of zinc compounds against *C. albicans* strains. The MIC of all zinc compounds used was 1.0 mM against ATCC 10231 and 18804 strains. However, in the ATCC 11006 strain, a higher concentration of zinc compounds was required to inhibit the growth.

**Table 7:** Minimum inhibitory concentration of zinc compounds against *Candida albicans* strains

Zinc compounds	MIC (mM)		
*n* = 6	*Candida albicans* strain ATCC 10231	*Candida albicans* strain ATCC 11006	*Candida albicans* strain ATCC 18804
Zinc chloride	1.0	2.4	1.0
Zinc gluconate	1.0	2.4	1.0
Zinc lactate	1.0	2.4	1.0
Zinc sulfate	1.0	2.0	1.0

*MIC* minimum inhibitory concentration

#### Candidacidal Activities of Zinc Compounds

Tables 8, 9, and 10 show the concentration-dependent candidacidal activities of zinc compounds. Zinc compounds showed candidacidal activities of varying degrees even at a concentration of 1 μM (ATCC 10231 strain, 10.7–18.6%; ATCC 11006 strain, 21.4–25.5%; and ATCC 18804 strain, 5.7–23.6%). For all three *C. albicans* strains used, the candidacidal activities of zinc compounds increased in concentration-dependent manner.

**Table 8:** Candidacidal activities of zinc compounds according to concentration (*Candida albicans* strain ATCC 10231)

Zinc compounds	*n* = 6	Control	1 μM	10 μM	100 μM	1 mM^§^	10 mM^§^
Zinc chloride	CFU	118.5 [108.4–131.5] (119.6)	106.3 [94.3–111.4] (103.7)	94.0 [80.4–103.9] (93.7)	88.8 [82.8–99.1] (90.3)	90.3 [87.5–95.6] (91.7)	88.3 [84.8–92.0] (87.9)
	% killing	–	15.3 [2.7–18.7] (12.9)	22.0 [4.8–34.3] (20.7)	23.8 [19.9–29.6] (24.3)	19.4 [15.5–31.2] (22.6)^a^	23.7 [19.1–33.5] (25.7)^a^
Zinc gluconate	CFU	131.8 [114.1–138.0] (129.3)	111.5 [96.8–119.1] (108.3)	105.3 [99.0–122.4] (109.2)	97.8 [88.8–102.3] (96.1)	98.0 [90.0–106.9] (98.6)	89.5 [76.6–102.0] (90.4)
	% killing	–	17.9 [3.2–27.5] (15.9)	13.4 [8.2–21.4] (15.1)	22.0 [18.6–36.6] (25.0)	21.9 [16.0–31.7] (23.3)^b^	29.0 [22.4–39.1] (29.8)^b^
Zinc lactate	CFU	123.5 [114.9–138.3] (126.9)	110.5 [104.5–112.6] (109.1)	100.8 [97.1–109.4] (102.8)	98.3 [83.1–110.3] (97.5)	95.3 [93.4–102.0] (97.7)	89.8 [86.4–94.5] (90.3)
	% killing	–	10.7 [5.5–21.2] (13.2)	18.8 [10.3–25.2] (18.3)	22.9 [16.1–32.4] (22.9)	17.7 [17.0–29.4] (22.3)^c^	24.3 [22.3–37.2] (28.1)^c^
Zinc sulfate	CFU	92.8 [85.6–99.3] (91.3)	69.0 [63.3–81.5] (71.0)	68.5 [58.8–77.0] (67.8)	62.3 [57.1–65.1] (61.1)	57.8 [56.6–59.8] (58.0)	51.8 [46.6–55.5] (51.3)
	% killing	–	18.6 [11.1–33.4] (21.6)	27.1 [14.7–35.7] (25.6)	33.3 [25.8–40.8] (32.4)	38.4 [30.8–40.2] (35.8)^a,b,c^	45.2 [38.2–47.5] (43.5)^a,b,c^

Median [interquartile range] (mean)

*CFU* colony-forming unit

The Kruskal–Wallis test was used to analyze differences in the ratio among zinc compounds at the same concentration. ^§^*P* < 0.05

The Mann–Whitney *U* test was used for post hoc analysis. Pairs of the same letter denote a significant difference at the same concentration. *P* < 0.05

**Table 9:** Candidacidal activities of zinc compounds according to concentration (*Candida albicans* strain ATCC 11006)

Zinc compounds	*n* = 6	Control	1 μM	10 μM^§^	100 μM	1 mM	10 mM^§^
Zinc chloride	CFU	117.5 [89.3–119.4] (107.4)	87.3 [66.4–96.4] (82.9)	87.5 [73.5–95.9] (84.6)	78.0 [70.5–80.0] (74.8)	85.5 [64.3–92.0] (78.5)	71.3 [54.8–79.9] (66.5)
	% killing	–	21.5 [19.3–27.5] (22.8)	20.2 [17.8–25.3] (21.1)^a^	31.5 [22.5–35.6] (29.5)	23.5 [16.9–42.1] (27.5)	36.4 [32.2–45.8] (39.1)
Zinc gluconate	CFU	99.3 [88.1–103.0] (96.3)	78.0 [70.9–82.1] (76.2)	75.3 [67.4–82.6] (74.4)	73.0 [63.0–82.4] (71.4)	63.3 [58.9–73.1] (65.6)	60.3 [55.1–65.9] (60.6)
	% killing	–	21.4 [17.5–24.6] (21.0)	23.4 [19.1–27.7] (23.0)	23.4 [20.6–34.6] (26.2)	29.4 [25.5–38.3] (31.6)	37.4 [33.0–41.3] (37.1)^a^
Zinc lactate	CFU	76.3 [68.9–94.9] (80.0)	58.5 [50.1–71.5] (60.3)	52.3 [50.8–65.8] (56.4)	45.3 [42.4–68.3] (52.0)	47.0 [45.5–57.9] (50.1)	36.8 [35.3–50.8] (41.1)
	% killing	–	24.7 [20.5–28.5] (24.6)	31.5 [22.8–33.8] (29.1)	37.0 [29.6–40.5] (35.5)	38.8 [31.6–41.0] (36.8)	47.0 [46.0–52.6] (48.7)^a,b^
Zinc sulfate	CFU	96.3 [85.9–104.9] (95.4)	73.0 [60.9–80.5] (71.6)	62.3 [57.6–75.8] (66.0)	66.5 [63.6–70.4] (67.3)	59.8 [55.5–68.9] (62.8)	55.3 [52.0–62.0] (56.4)
	% killing	–	25.5 [22.1–28.3] (25.1)	30.5 [24.5–36.1] (30.9)^a^	29.4 [23.8–35.3] (29.1)	35.7 [24.4–43.0] (33.8)	42.4 [34.6–44.5] (40.5)^b^

Median [interquartile range] (mean)

*CFU* colony-forming unit

The Kruskal–Wallis test was used to analyze differences in the ratio among zinc compounds at the same concentration. ^§^*P* < 0.05

The Mann–Whitney *U* test was used for post hoc analysis. Pairs of the same letter denote a significant difference at the same concentration. *P* < 0.05

**Table 10:** Candidacidal activities of zinc compounds according to concentration (*Candida albicans* strain ATCC 18804)

Zinc compounds	*n* = 6	Control	1 μM^§^	10 μM	100 μM	1 mM^§^	10 mM^§^
Zinc chloride	CFU	155.8 [127.1–168.1] (150.3)	129.6 [115.2–157.1] (134.4)	126.5 [88.9–140.7] (118.2)	112.5 [95.0–131.9] (112.3)	111.5 [96.3–121.4] (109.3)	102.8 [92.6–112.6] (102.9)
	% killing	–	11.4 [6.0–16.3] (10.8)^a^	21.9 [10.3–33.1] (21.9)	23.4 [21.4–31.9] (25.6)	26.3 [19.9–34.5] (27.0)	32.5 [23.0–38.7] (31.4)^a^
Zinc gluconate	CFU	138.0 [120.6–165.9] (142.0)	130.3 [107.5–160.0] (133.0)	115.5 [88.5–124.7] (108.4)	106.0 [92.9–119.1] (106.7)	114.5 [109.6–123.3] (116.4)	98.5 [87.0–106.5] (98.0)
	% killing	–	5.7 [1.5–10.3] (6.4)^b^	18.4 [13.0–38.5] (22.9)	24.9 [5.4–38.3] (22.6)	18.5 [2.9–27.9] (16.3)^a^	30.7 [6.8–47.9] (28.4)
Zinc lactate	CFU	140.8 [121.6–164.5] (142.3)	128.0 [115.3–153.6] (132.1)	118.3 [98.1–130.7] (116.1)	107.3 [101.5–126.1] (111.0)	108.0 [96.8–135.1] (113.8)	119.5 [88.3–126.3] (111.5)
	% killing	–	7.0 [2.3–11.3] (6.9)^c^	16.9 [11.5–25.6] (17.9)	18.5 [14.2–26.0] (21.0)	20.0 [11.8–24.6] (19.8)^b^	24.8 [15.5–26.2] (21.7)^b^
Zinc sulfate	CFU	138.5 [131.8–143.4] (137.4)	104.5 [97.0–111.3] (105.3)	93.5 [91.0–97.4] (94.6)	90.3 [81.5–95.1] (88.2)	92.8 [82.9–95.6] (89.8)	79.0 [72.1–85.3] (78.4)
	% killing	–	23.6 [14.9–32.6] (23.2)^a,b,c^	31.5 [26.1–34.8] (31.0)	34.0 [30.6–40.5] (35.7)	32.5 [29.7–39.4] (34.5)^a,b^	43.8 [35.8–49.6] (42.6)^a,b^

Median [interquartile range] (mean)

*CFU* colony-forming unit

The Kruskal–Wallis test was used to analyze differences in the ratio among zinc compounds at the same concentration. ^§^*P* < 0.05

The Mann–Whitney *U* test was used for post hoc analysis. Pairs of the same letter denote a significant difference at the same concentration. *P* < 0.05

In all three strains, candidacidal activities differed significantly according to the type of zinc compounds. These differences were mainly noticeable at the highest concentration (10 mM). In the ATCC 10231 and 18804 strains, zinc sulfate exhibited the highest candidacidal activity (45.2% and 43.8%, respectively), whereas in the ATCC 11006 strain, zinc lactate showed the highest candidacidal activity (47.0%).

The candidacidal activities of zinc chloride at concentrations of 1 μM and 10 mM and zinc lactate at concentrations of 1 μM, 100 μM, 1 mM, and 10 mM differed significantly according to the *C. albicans* strain (*P* < 0.05). Both zinc compounds showed higher candidacidal activity against ATCC strain 11006 (data not shown).

## Discussion

This study was conducted to determine the effects of zinc compounds on the activities of salivary antimicrobial enzymes and their antifungal activity. The results demonstrated that zinc is a promising component for oral health care products. Zinc compounds increased the enzymatic activities of salivary lysozyme in solution and of HEWL and salivary lysozyme on the hydroxyapatite surface. In contrast, zinc compounds inhibited the enzymatic activities of bLPO and salivary peroxidase, in solution and on the surface. Direct binding of zinc compounds to lysozyme and peroxidase molecules was observed. Zinc compounds exhibited concentration-dependent candidacidal activity against *C. albicans*.

Several studies have reported the effects of zinc compounds on the enzymatic activities of lysozyme and peroxidase derived from bacteria, fungi, plants, and animals [32–39]. However, only one clinical study reported insignificant effects of dentifrices containing zinc citrate on the levels of human salivary lysozyme and peroxidase [40]. No study has reported the direct effects of zinc compounds on the enzymatic activities of human salivary lysozyme and peroxidase, and on hydroxyapatite surfaces. This is the first study to show the effects of zinc compounds on lysozyme and peroxidase in human saliva and on hydroxyapatite surfaces.

We found that zinc compounds, especially zinc gluconate and sulfate, increased the enzymatic activities of salivary lysozyme in solution and of HEWL and salivary lysozyme on the surface. However, previous studies have conflicting reports of zinc inhibiting lysozyme activity [32, 33]. One of such studies used glucosaminidase instead of a true lysozyme substance [32]. Another study used starfish lysozyme; therefore, the results could be the metabolic effect of zinc rather than a direct interaction of zinc with lysozyme [33]. Although the exact mechanism of zinc compounds affecting lysozyme activity remains unclear, it has been reported that zinc ion binds to glycoside and catalyzes hydrolysis of glycoside bonds [41, 42], which could explain the additive effects of zinc compounds on lysozyme activity. The interactions among zinc compounds, salivary lysozyme, and other molecules in human saliva, such as mucins, could explain the different effects of zinc between HEWL and salivary lysozyme in the solution assay [24, 30]. The conformational changes in the lysozyme molecules on the surface could explain the difference in the effects of zinc on HEWL between solution and surface assays [21–23, 43].

The results of significant inhibition of the enzymatic activities of bLPO and salivary peroxidase by zinc compounds were consistent with previously published studies using fungal, plant, and animal-derived peroxidases [34–39]. The more pronounced effects on the surface rather than in solution, with noticeable effects on salivary peroxidase than bLPO, could be explained by the same way as lysozyme: conformational changes on the surface and interactions with other salivary molecules, respectively. Given that zinc exhibits different activities on cationic and anionic peroxidase isozymes, the mechanism for the effect of zinc on peroxidase could be an ionic interaction [36]. The inhibitory effects of zinc on HRP occurring in a noncompetitive pattern have been reported [38]. Salivary peroxidase and LPO are heme-containing proteins whose active site has a ferric ion (Fe^3+^)-containing porphyrin molecule. Anions including chloride or sulfate could be coordinated to the iron-binding porphyrin. Therefore, the disturbances of peroxidase activity could be caused by the coordination of counter anions in zinc compounds toward iron-binding porphyrin of peroxidase [44].

The results of SPR analyses revealed direct binding of zinc compounds to HEWL and HRP and that zinc chloride had a higher affinity than zinc sulfate. In an aqueous solution, two moles of chloride ions and one mole of sulfate ions were generated. The concentration difference in anions could affect the binding affinity to proteins. In addition, the hydrolysis constants of zinc chloride and sulfate may affect their protein bindings [45]. The hydrolysis constant of zinc chloride is higher than that of zinc sulfate, which means that zinc chloride is easy to ionize comparing to zinc sulfate.

Although the antibacterial activity of zinc compounds has been reported [5–7], their antifungal activity has been less studied. Some studies have reported fungal growth inhibition and fungicidal activity by zinc compounds, but did not target oral fungi but rather fungi involved in grain deterioration [9] and plant diseases [17]. Another study on antifungal activity as a supplement to antibacterial activity used one *C. albicans* strain and reported only MIC data of zinc gluconate [7]. In the present study, the reported MIC values of four zinc compounds against three *C. albicans* strains were lower than the zinc compound concentrations used in studies on the development of oral health care products [6, 13, 16, 29]. Based on the reported candidacidal activities at concentrations of 1–10 mM, the present study suggests that zinc compounds, especially zinc sulfate, could be developed as potential topical antifungal agents for the prevention or treatment of oral candidiasis. However, there are few studies on the underlying mechanisms of the antifungal effects of zinc compounds. In a study using food-related fungi, zinc compounds affected conidia production, hyphae morphological alterations, and mortality [9]. Therefore, further studies on antifungal mechanisms targeting oral *Candida* species are required.

Although zinc is a relatively safe substance, toxicity issues need to be considered. The frequent reports related to zinc toxicity to human health were gastrointestinal symptoms due to excessive consumption, which also needs to be considered for the development of topical agents in the oral cavity [46]. The oxidant/antioxidant effects of zinc also need to be considered. The oxidation effect of zinc ions is not carried out by zinc alone, but zinc can participate in oxidation reaction by binding zinc with susceptible ligands. In addition, in the redox signal pathway caused by zinc, zinc plays a role in forming a disulfide bond by combining with the thiol of cysteine ​residue of proteins without changing the oxidation state of zinc [47]. The antioxidant effect of zinc is well-known. The antioxidant effects of zinc are mediated through inhibiting the production of reactive oxygen species, its binding to thiol groups, its structural role in antioxidant proteins, and modulation metallothionein induction [48].

With the elderly population growing at an unprecedented pace, decreased salivation and xerostomic symptoms are becoming very common, increasing the incidence of geriatric oral diseases, including oral candidiasis, cervical caries, gingivitis/periodontitis, oral ulcers, and oral malodor. Therefore, it is necessary to have oral health care products that can effectively and safely address these problems. Of four zinc compounds examined in this study, zinc sulfate was found to be the most useful compound in terms of lysozyme and antifungal activities. However, further research is warranted to overcome the inhibition of peroxidase activity by zinc compounds.

## Conclusions

Zinc compounds enhanced lysozyme activity but inhibited peroxidase activity, and showed concentration-dependent candidacidal activity against *C. albicans*. Considering the known beneficial biological properties of zinc for oral health and the results of this study, zinc compounds could be used to develop effective topical therapeutic agents, especially for common oral sufferings of geriatric patients. To achieve these goals, further research, including toxic effects, compositions, and advanced clinical studies, is warranted.

## Supplementary Information

Below is the link to the electronic supplementary material.

Supplementary file1 (DOCX 39 KB)

## Data Availability

The data that support the findings of this study are available from the corresponding author upon reasonable request.
