# Supplementary material for: Effects of Zinc Compounds on the Enzymatic Activities of Lysozyme and Peroxidase and Their Antifungal Activities
Source: Biol Trace Elem Res. 2024 Feb 20;202(12):5850–62. doi: 10.1007/s12011-024-04110-x (PMC11502591; doi:10.1007/s12011-024-04110-x)
Supplement: Supplementary file 1 — Supplementary file1 (DOCX 39 KB) [file 12011_2024_4110_MOESM1_ESM.docx]

| Source of lysozyme | n = 8 | Lysozyme activity (Unit/mL) |  |  |  |  |  |  |  |  |  |  |  |  |  |  |  |
| --- | --- | --- | --- | --- | --- | --- | --- | --- | --- | --- | --- | --- | --- | --- | --- | --- | --- |
|  | Zinc  (mM) | Zinc chloride |  |  |  | Zinc gluconate |  |  |  | Zinc lactate |  |  |  | Zinc sulfate |  |  |  |
|  |  | Sample only | With zinc | Ratio (%) | *P* value | Sample only | With zinc | Ratio (%) | *P* value | Sample only | With zinc | Ratio (%) | *P* value | Sample only | With zinc | Ratio (%) | *P* value |
| HEWL | 3.6 | 2164.8 [2004.9 – 2416.1] (2159.7) | 2219.4 [2095.1 – 2458.9] (2207.4) | 102.1 [99.3 – 104.8] (102.3) | .093 | 2579.2 [2505.4 – 2728.4] (2598.9) | 2588.4 [2551.4 – 2751.6] (2628.7) | 100.7 [98.6 – 104.6] (101.2) | .401 | 1966.1 [1813.3 – 2125.6] (1953.1) | 2015.2 [1843.1 – 2090.6] (1976.8) | 101.4 [99.7 – 103.8] (101.4) | .208 | 2558.5 [2500.3 – 2607.9] (2566.9) | 2462.8 [2406.2 – 2600.7] (2512.3) | 96.2 [94.6 – 101.2] (97.9) | .263 |
|  | 7.2 | 2132.2 [1953.4 – 2405.3] (2173.4) | 2206.8 [1945.7 – 2501.1] (2227.5) | 104.2 [97.8 – 105.7] (102.3) | .093 | 2843.5 [2624.0 – 2874.6] (2788.6) | 2751.9 [2613.6 – 2864.1] (2745.9) | 98.7 [95.9 – 100.0] (98.5) | .123 | 1871.6 [1714.3 – 2022.7] (1879.0) | 1873.3 [1801.1 – 2022.8] (1906.9) | 100.8 [99.7 – 104.6] (101.6) | .263 | 2531.3 [2403.8 – 2638.3] (2528.1) | 2498.0 [2427.5 – 2577.3] (2505.0) | 98.9 [96.9 – 101.7] (99.1) | .327 |
| Human saliva | 3.6^§^ | 670.7 [594.8 – 725.2] (663.5) | 675.3 [619.3 – 751.1] (674.7) | 101.3 [99.4 – 105.5] (101.8)^a,b^ | .263 | 544.4 [434.9 – 566.3] (513.6) | 606.3 [481.0 – 640.7] (572.3) | 111.3 [108.8 – 114.2] (111.5)^a,c,d^ | .012* | 600.2 [571.2 – 629.8] (598.2) | 608.2 [590.9 – 631.4] (606.9) | 101.4 [98.7 – 104.6] (101.6)^c,e^ | .208 | 380.0 [331.3 – 442.6] (387.2) | 514.5 [469.4 – 550.6] (512.4) | 130.6 [124.1 – 146.9] (133.9)^b,d,e^ | .012* |
|  | 7.2^§^ | 660.2 [587.2 – 728.7] (660.6) | 652.9 [598.3 – 741.1] (664.7) | 101.6 [100.6 – 102.6] (100.6)^a,b^ | .161 | 564.6 [456.3 – 590.3] (528.0) | 631.7 [539.5 – 669.8] (606.4) | 118.3 [106.5 – 122.6] (115.3)^a,c,d^ | .012* | 541.9 [517.1 – 592.5] (554.0) | 563.3 [527.3 – 608.7] (567.3) | 102.9 [100.1 – 104.5] (102.5)^c,e^ | .050 | 374.9 [331.6 – 426.5] (377.0) | 579.8 [564.3 – 592.0] (573.7) | 154.0 [138.8 – 167.3] (153.8)^b,d,e^ | .012* |

Supplementary Table 1.1. Effect of zinc compounds on the enzymatic activities of hen egg-white lysozyme and whole salivary lysozyme in solution

Median [interquartile range] (mean)

HEWL, hen egg-white lysozyme.

Ratio, percent changes in enzymatic activities in samples with zinc compounds compared with those without zinc compounds.

The Wilcoxon signed rank test was used to analyze differences between the enzymatic activities of samples with and without zinc compounds. **P* < 0.05.

The Kruskal-Wallis test was used to analyze differences in the ratio among zinc compounds at the same concentration. ^§^*P* < 0.05.

The Mann–Whitney U test was used for post-hoc analysis. Pairs of the same letter denote a significant difference at the same concentration. *P* < 0.05.

Supplementary Table 1.2. Effect of zinc compounds on the enzymatic activities of hen egg-white lysozyme and whole salivary lysozyme on the hydroxyapatite surface

| Source of lysozyme | n = 8 | Lysozyme activity (Units) |  |  |  |  |  |  |  |  |  |  |  |  |  |  |  |
| --- | --- | --- | --- | --- | --- | --- | --- | --- | --- | --- | --- | --- | --- | --- | --- | --- | --- |
|  | Zinc (mM) | Zinc chloride |  |  |  | Zinc gluconate |  |  |  | Zinc lactate |  |  |  | Zinc sulfate |  |  |  |
|  |  | Sample only | With zinc | Ratio (%) | *P* value | Sample only | With zinc | Ratio (%) | *P* value | Sample only | With zinc | Ratio (%) | *P* value | Sample only | With zinc | Ratio (%) | *P* value |
| HEWL | 3.6^§^ | 65.0 [62.3 – 66.5] (64.3) | 63.4 [61.0 – 66.5] (63.7) | 98.8 [98.3 – 101.4] (99.0)^a,b^ | .484 | 50.3 [46.7 – 55.8] (51.2) | 53.5 [48.5 – 57.0] (53.8) | 105.7 [103.8 – 107.7] (105.1)^a,c,d^ | .017* | 60.1 [58.8 – 60.9] (60.0) | 60.1 [58.6 – 60.5] (59.7) | 99.7 [98.1 – 102.0] (99.6)^c,e^ | .779 | 53.3 [49.7 – 56.6] (52.5) | 60.5 [56.5 – 62.7] (59.5) | 113.9 [110.1 – 115.5] (113.4)^b,d,e^ | .012* |
|  | 7.2^§^ | 71.5 [60.1 – 76.5] (69.0) | 73.5 [67.0 – 82.7] (74.0) | 108.3 [103.0 – 111.4] (107.5)^a^ | .017* | 48.0 [45.5 – 54.1] (49.5) | 49.9 [47.3 – 58.6] (52.4) | 104.2 [101.8 – 109.8] (105.6)^b^ | .012* | 56.9 [56.7 – 59.7] (57.8) | 60.7 [59.3 – 62.8] (60.7) | 103.8 [100.6 – 110.7] (105.2)^c^ | .012* | 52.6 [44.0 – 59.2] (52.3) | 61.4 [52.8 – 72.0] (62.3) | 119.2 [116.4 – 121.2] (119.0)^a,b,c^ | .012* |
| Human saliva | 3.6^§^ | 33.1 [29.9 – 35.5] (32.6) | 33.1 [27.3 – 35.4] (31.9) | 96.0 [93.3 – 101.9] (97.6)^a,b^ | .263 | 33.9 [31.8 – 34.8] (33.4) | 37.5 [34.8 – 40.0] (37.0) | 108.7 [104.1 – 120.1] (111.0)^a^ | .012* | 26.9 [23.0 – 29.1] (26.0) | 25.7 [24.7 – 26.8] (26.1) | 102.0 [91.1 – 110.3] (101.1)^c^ | .889 | 24.9 [20.7 – 26.3] (24.1) | 28.1 [27.4 – 32.3] (29.3) | 125.0 [109.5 – 134.9] (122.5)^b,c^ | .017* |
|  | 7.2^§^ | 34.7 [31.4 – 38.4] (34.6) | 35.0 [31.2 – 38.0] (34.5) | 97.0 [95.9 – 99.8] (100.1)^a,b^ | .208 | 34.7 [29.7 – 37.4] (34.0) | 42.0 [35.7 – 43.7] (40.3) | 117.8 [107.2 – 133.2] (119.6)^a,c,d^ | .012* | 25.4 [24.0 - 27.8] (25.9) | 26.5 [24.7 – 30.7] (27.6) | 106.0 [102.0 – 109.2] (106.1)^c^ | .046* | 22.4 [20.7 – 24.7] (22.9) | 28.2 [25.3 – 31.3] (28.9) | 125.3 [123.0 – 130.7] (125.7)^b,d^ | .012* |

Median [interquartile range] (mean)

HEWL, hen egg-white lysozyme.

Ratio, percent changes in enzymatic activities in samples with zinc compounds compared with those without zinc compounds.

The Wilcoxon signed rank test was used to analyze differences between the enzymatic activities of samples with and without zinc compounds. **P* < 0.05.

The Kruskal-Wallis test was used to analyze differences in the ratio among zinc compounds at the same concentration. ^§^*P* < 0.05.

The Mann–Whitney U test was used for post-hoc analysis. Pairs of the same letter denote a significant difference at the same concentration. *P* < 0.05.

Supplementary Table 2.1. Effect of zinc compounds on the enzymatic activities of bovine lactoperoxidase and whole salivary peroxidase in solution

| Source of peroxidase | n = 8 | Peroxidase activity (Unit/mL) |  |  |  |  |  |  |  |  |  |  |  |  |  |  |  |
| --- | --- | --- | --- | --- | --- | --- | --- | --- | --- | --- | --- | --- | --- | --- | --- | --- | --- |
|  | Zinc (mM) | Zinc chloride |  |  |  | Zinc gluconate |  |  |  | Zinc lactate |  |  |  | Zinc sulfate |  |  |  |
|  |  | Sample only | With zinc | Ratio (%) | *P* value | Sample only | With zinc | Ratio (%) | *P* value | Sample only | With zinc | Ratio (%) | *P* value | Sample only | With zinc | Ratio (%) | *P* value |
| bLPO | 3.6 | 5.98 [5.63 – 8.68] (6.89) | 5.88 [5.49 – 7.71] (6.43) | 93.1 [89.7 – 97.6] (93.9)^a^ | .021* | 7.29 [5.73 – 7.74] (6.92) | 6.52 [5.50 – 6.93] (6.29) | 89.6 [85.7 – 98.5] (91.6)^b^ | .036* | 8.73 [8.13 – 9.06] (8.64) | 7.71 [7.50 – 8.28] (7.94) | 95.1 [90.9 – 95.7] (93.9) | .050 | 6.01 [5.43 – 6.61] (6.01) | 6.08 [5.44 – 6.28] (5.98) | 102.2 [94.3 – 103.0] (99.5)^a,b^ | .674 |
|  | 7.2 | 7.26 [4.90 – 8.05] (6.73) | 5.87 [4.43 – 7.10] (5.87) | 93.7 [73.8 – 98.2] (88.7) | .012* | 6.48 [6.02 – 6.79] (6.40) | 5.42 [5.08 – 5.70] (5.48) | 87.1 [74.8 – 96.2] (86.4) | .025* | 8.84 [8.68 – 9.02] (8.88) | 7.64 [7.00 – 8.10] (7.58) | 86.3 [78.7 – 90.3] (85.4) | .012* | 6.01 [5.79 – 6.68] (6.28) | 5.83 [5.46 – 6.21] (5.92) | 93.3 [92.2 – 97.1] (94.4) | .012* |
| Human saliva | 3.6^§^ | 0.33 [0.32 – 0.38] (0.35) | 0.28 [0.27 – 0.32] (0.29) | 84.4 [83.7 – 85.9] (84.6)^a^ | .012* | 0.41 [0.40 – 0.42] (0.41) | 0.38 [0.37 – 0.40] (0.38) | 93.1 [89.1 – 94.8] (92.5)^a,b,c^ | .012* | 0.38 [0.30 – 0.41] (0.36) | 0.34 [0.26 – 0.34] (0.31) | 85.7 [84.9 – 87.4] (86.0)^b^ | .012* | 0.38 [0.37 – 0.41] (0.39) | 0.33 [0.32 – 0.35] (0.33) | 86.6 [82.4 – 89.0] (85.9)^c^ | .012* |
|  | 7.2^§^ | 0.32 [0.31 – 0.35] (0.33) | 0.26 [0.24 – 0.26] (0.25) | 76.2 [75.0 – 80.4] (76.9)^a,b^ | .012* | 0.40 [0.39 – 0.41] (0.39) | 0.35 [0.35 – 0.36] (0.35) | 89.0 [86.5 – 90.6] (88.5)^a,c,d^ | .012* | 0.37 [0.28 – 0.38] (0.35) | 0.30 [0.23 – 0.32] (0.28) | 82.1 [79.7 – 83.4] (81.5)^b,c,e^ | .012* | 0.40 [0.38 – 0.43] (0.41) | 0.32 [0.30 – 0.33] (0.31) | 75.4 [74.3 – 81.4] (77.2)^d,e^ | .012* |

Median [interquartile range] (mean)

bLPO, bovine lactoperoxidase.

Ratio, percent changes in enzymatic activities in samples with zinc compounds compared with those without zinc compounds.

The Wilcoxon signed rank test was used to analyze differences between the enzymatic activities of samples with and without zinc compounds. **P* < 0.05.

The Kruskal-Wallis test was used to analyze differences in the ratio among zinc compounds at the same concentration. ^§^*P* < 0.05.

The Mann–Whitney U test was used for post-hoc analysis. Pairs of the same letter denote a significant difference at the same concentration. *P* < 0.05.

Supplementary Table 2.2. Effect of zinc compounds on the enzymatic activities of bovine lactoperoxidase and whole salivary peroxidase on the hydroxyapatite surface

| Source of peroxidase | n = 8 | Peroxidase activity (mUnits) |  |  |  |  |  |  |  |  |  |  |  |  |  |  |  |
| --- | --- | --- | --- | --- | --- | --- | --- | --- | --- | --- | --- | --- | --- | --- | --- | --- | --- |
|  | Zinc (mM) | Zinc chloride |  |  |  | Zinc gluconate |  |  |  | Zinc lactate |  |  |  | Zinc sulfate |  |  |  |
|  |  | Sample only | With zinc | Ratio (%) | *P* value | Sample only | With zinc | Ratio (%) | *P* value | Sample only | With zinc | Ratio (%) | *P* value | Sample only | With zinc | Ratio (%) | *P* value |
| bLPO | 3.6^§^ | 4.03 [3.93 – 4.22] (4.41) | 3.85 [3.78 – 4.03] (4.20) | 95.0 [94.9 – 96.6] (95.4)^a,b^ | .012* | 5.28 [5.14 – 5.47] (5.32) | 5.41 [5.13 – 5.61] (5.38) | 101.8 [96.2 – 105.3] (101.3)^a^ | .327 | 5.39 [5.09 – 5.76] (5.39) | 5.20 [4.92 – 5.44] (5.21) | 96.5 [96.0 – 97.6] (96.6)^c^ | .011* | 4.27 [3.95 – 4.75] (4.31) | 4.21 [4.12 – 4.74] (4.37) | 102.5 [97.4 – 104.9] (101.5)^b,c^ | .484 |
|  | 7.2^§^ | 4.00 [3.50 – 4.12] (3.87) | 3.48 [3.26 – 3.57] (3.42) | 88.1 [86.0 – 91.7] (88.7)^a,b^ | .012* | 5.40 [5.22 – 5.56] (5.40) | 5.15 [4.95 – 5.54] (5.23) | 97.3 [94.7 – 99.3] (96.7)^a,c,d^ | .017* | 5.07 [4.89 – 5.77] (5.29) | 4.83 [4.73 – 5.38] (5.00) | 93.8 [93.2 – 96.7] (94.7)^b,c^ | .012* | 4.94 [4.47 – 5.24] (4.92) | 4.46 [4.32 – 4.90] (4.58) | 93.5 [88.8 – 97.6] (93.4)^d^ | .025* |
| Human saliva | 3.6 | 6.73 [6.20 – 7.24] (6.72) | 3.55 [3.37 – 4.04] (3.66) | 54.1 [47.4 – 61.7] (54.8) | .012* | 7.91 [7.71 – 8.70] (8.17) | 4.87 [4.36 – 5.28] (4.84) | 60.1 [56.7 – 61.1] (59.1) | .012* | 7.30 [7.08 – 7.59] (7.33) | 4.15 [3.83 – 4.76] (4.24) | 56.1 [53.4 – 62.6] (57.8) | .012* | 7.49 [7.23 – 7.94] (7.56) | 4.03 [3.91 – 4.56] (4.19) | 55.1 [53.7 – 57.2] (55.4) | .012* |
|  | 7.2^§^ | 6.42 [5.96 – 7.03] (6.46) | 2.75 [2.52 – 3.37] (2.90) | 44.7 [40.3 – 49.1] (45.0)^a,b,c^ | .012* | 8.36 [7.56 – 8.50] (8.14) | 3.63 [3.30 – 4.10] (3.74) | 45.8 [43.2 – 48.3] (46.0)^a^ | .012* | 7.27 [7.07 – 7.39] (7.18) | 3.20 [2.86 – 3.74] (3.29) | 44.0 [39.1 – 52.9] (45.7)^b^ | .012* | 8.31 [7.86 – 8.84] (8.32) | 3.66 [3.13 – 4.05] (3.63) | 42.3 [39.5 – 48.4] (43.5)^c^ | .012* |

Median [interquartile range] (mean)

bLPO, bovine lactoperoxidase.

Ratio, percent changes in enzymatic activities in samples with zinc compounds compared with those without zinc compounds.

The Wilcoxon signed rank test was used to analyze differences between the enzymatic activities of samples with and without zinc compounds. **P* < 0.05.

The Kruskal-Wallis test was used to analyze differences in the ratio among zinc compounds at the same concentration. ^§^*P* < 0.05.

The Mann–Whitney U test was used for post-hoc analysis. Pairs of the same letter denote a significant difference at the same concentration. *P* < 0.05.

Supplementary Table 3. Effect of zinc compounds on the enzymatic activities of glucose oxidase-mediated peroxidase

| Zinc compounds | Enzymatic activity of glucose oxidase-mediated peroxidase (n = 5) | | | | | | |
| --- | --- | --- | --- | --- | --- | --- | --- |
|  | Sample only | With zinc at 3.6 mM^§^ | | | With zinc at 7.2 mM^§^ | | |
|  | OD | OD | Ratio (%) | *P* value | OD | Ratio (%) | *P* value |
| Zinc chloride | .683 [.658 – .701] (.680) | .686 [.640 – .696] (.671) | 98.3 [97.2 – 100.4] (98.7)^a^ | .225 | .685 [.658 – .706] (.683) | 100.3 [100.0 – 100.6] (100.3)^a^ | .144 |
| Zinc gluconate | .715 [.712 – .730] (.720) | .725 [.723 – .729] (.726) | 101.4 [99.7 – 101.8] (100.9) | .225 | .725 [.724 – .729] (.726) | 101.3 [99.9 – 101.7] (100.9)^b^ | .138 |
| Zinc lactate | .723 [.712 – .744] (.727) | .721 [.704 – .738] (.721) | 99.6 [98.3 – 99.9] (99.2) | .068 | .720 [.705 - .732] (.719) | 99.2 [98.0 – 99.7] (98.9)^a,b,c^ | .043* |
| Zinc sulfate | .705 [.701 - .718] (.708) | .711 [.709 - .717] (.712) | 100.9 [99.9 – 101.1] (100.6)^a^ | .138 | .713 [.708 - .727] (.716) | 101.1 [100.2 – 102.0] (101.1)^c^ | .078 |

Median [interquartile range] (mean)

OD, optical density

The Wilcoxon signed rank test was used to analyze differences between the enzymatic activities of samples with and without zinc compounds. **P* < 0.05.

The Kruskal-Wallis test was used to analyze differences in the ratio among zinc compounds at the same concentration. ^§^*P* < 0.05.

The Mann–Whitney U test was used for post-hoc analysis. Pairs of the same letter denote a significant difference at the same concentration. *P* < 0.05
